# Apparent diffusion coefficient as an effective index for the therapeutic efficiency of brain chemoradiotherapy for brain metastases from lung cancer

**DOI:** 10.1186/s12880-018-0275-3

**Published:** 2018-09-17

**Authors:** Kai Liu, Zenglin Ma, Lili Feng

**Affiliations:** Department of Radiology, The Third Affiliated Hospital of Beijing University of Chinese Medicine, No. 51 Xiaoguan Street, Andingmenwai, Chaoyang District, Beijing, People’s Republic of China

**Keywords:** Apparent diffusion coefficient (ADC), Magnetic resonance imaging (MRI), Brain metastases, Therapeutic efficiency

## Abstract

**Background:**

The potential of apparent diffusion coefficient (ADC) value alteration before and after chemoradiotherapy as a potential monitor for therapeutic efficiency of treatment for brain metastases from lung cancer were discussed.

**Method:**

Thirty lung cancer patients with brain metastases, conventional magnetic resonance imaging (MRI) examination and diffusion weighted imaging (DWI) were performed one week before chemoradiotherapy and after one treatment cycle and two treatment cycles. 43 tumor lesions were divided into effective group and invalid group according to the changes of the tumor size. The differences in ADC values at different time points before and after treatment in each treatment group were analyzed.

**Result:**

The maximum diameter of the tumor was no difference after one treatment cycle, but decreased after two treatment cycles. ADC values significantly increased after both one and two treatment cycles. In effective group, the ADC values were significantly increased after one and two treatment cycles. While, there are no difference in invalid group after one treatment cycle but decreased after two treatment cycles. ΔADC values in effective group after one and two treatment cycles were both significantly higher than those in the invalid group. ROC curve analysis then revealed that the area under the curve (AUC) of ΔADC after one treatment was 0.872.

**Conclusion:**

ADC values in brain metastases from lung cancer can help monitor and dynamically observe the therapeutic efficiency of whole brain chemoradiotherapy.

## Background

Brain metastases from are an increasingly common problem with an incidence ten times that of primary brain tumors and the diagnosis is often made by general radiologists [1, 2]. Cumulative incidence of brain metastases is noted to be particularly high for patients with locally advanced lung cancer and HER2 positive metastatic breast cancer, with figures of up to 30–60% reported [3–5].

Traditionally, magnetic resonance imaging (MRI) has been regarded as a gold standard for detection of brain metastases and used for assessing the site and number of metastases, planning for surgery or radiosurgery and assessing the response to therapy [6, 7]. However, the differentiation of high-grade gliomas from brain metastases represents a common differential diagnosis problem since both tumors may display similar imaging characteristics and contrast enhancement patters on conventional MRI [8]. Besides, it is not universally given to cancer patients who lack neurological symptoms at presentation. Even in patients with a normal MRI brain scan at diagnosis and limited-stage small cell lung cancer, a recent study found that 32.5% subsequently developed MRI evidence of brain metastases by the end of chemotherapy, before receiving prophylactic cranial irradiation [5]. In the course of radiotherapy for brain metastases from lung cancer, the changes of the tumor size exist significantly behind changes in biological and molecular levels. Thus, developing early biological indicators to measure radiotherapy efficacy has become an urgent need.

Newer MRI techniques have been applied to help diagnosis and prognosis. Diffusion-weighted magnetic resonance (MR) imaging detects the water mobility to reflect the morphologic and physiologic changes in tissues which was wildly applied in chest tumors characterization [9, 10]. tThe apparent diffusion coefficient (ADC) of the lesion but these readings have more recently been suggested to have some prognostic value as well as diagnostic in brain metastases cancers [11, 12]. An inverse relationship was observed between ADC values and tumor cellularity [8]. Previous studies had shown that ADC values can be used in differentiating between certain types of cerebral tumors [13–15]. However, the efficiency of the ADC values as an index of brain metastases, and its advantages over MRI remain controversial.

In the present study, we attempted to analyze the dynamic trends of maximal tumor diameter and ADC values at different time points after chemotherapy and whole-brain radiotherapy in patients with brain metastasis from lung cancer, to predict and to monitor the efficacy of ΔADC values as an index for brain metastasis from lung cancer after brain radiation therapy.

## Methods

### Patients

Institutional review board approval was obtained for this study. All the subjects were informed of the contents of the test and signed the informed consent voluntarily. From October 2013 to August 2016, 30 patients diagnosed with brain metastasis from lung cancer by pathology or clinical follow-up and clinical imaging tests were involved in the present study. The clinical characteristics of the 30 patients were shown in Table 1.

**Table 1:** The clinical characteristics of enrolled patients

Clinical characteristics	Case number
Lesion number	43
Mean age	63.6 (45–85)
Gender	Female 18: Male 12
Pathological type	
Squamous carcinoma	12
Adenocarcinoma	11
Small cell lung cancer	3
Undifferentiated carcinoma	4
Location of brain metastasis	
Frontal lobe	9
Parietal lobe	11
Occipital lobe	12
Basal ganglia	4
Opisthencephalon	4
Temporal lobe	2
Brainstem	1

Case inclusion criteria: 1. Patients who were pathologically diagnosed with brain metastases from lung cancer, no limitation of gender and ethnicity; 2. Patients who were receiving radiotherapy and chemotherapy for the first time to ensure the uniformity of the initial state; 3. Patients with at least one measurable lesion (necrosis and cystic lesions not included) in the parenchyma of the brain, lesion diameter > 10 mm.

Case exclusion criteria: 1. Patients who could not complete the entire treatment plan, the radiochemotherapy and Chinese medicine treatment were terminated in advance; 2. Patients who received pacemaker implantation, coronary intervention, and Coronary Artery Bypass Grafting (CABG), thus contraindicated to MRI; 3. Patients who were unable to cooperate with MRI examinations.

### Treatment protocols

Chemotherapy: oral administration of drugs that can pass through the blood-brain barrier, such as CCNU or me-CCNU, simultaneously supplemented by intramuscular injection of dexamethasone (MTX) once a week, 4 times as a treatment course, and the dehydration agent. Systemic chemotherapy is based on the patient’s condition, the primary lesion and the cellular type of the cancer: for adenocarcinoma, CMF regimen (cyclophosphamide (CTX), MTX, fluorouracil (5-FU)) or CAF regimen (CTX, M (ADM), 5-FU] was used; for squamous carcinoma, DAV regimen (cisplatin (DDP), ADM, vincristine (VCR)) was used; for undifferentiated cancer, CVA regimen (CTX, VCR, ADM) was used. The above treatment was conducted once every 4 weeks, each time as a treatment cycle. Radiotherapy: Cobalt-60 bilateral whole brain irradiation was conducted, the dosage was 4000-5000Gy, supplemented by mannitol dehydration or hormone therapy to reduce the radiotherapy response. Chinese traditional medicine: dialectical therapy.

### Examination protocols

All examinations were performed on Achieva 1.5 T SE MRI systems with an NV-8 combined coil for head and neck (Philips Healthcare, Amsterdam, Netherlands). The examinations were conducted with a gradient set of strength of 33 mT/m and a gradient switching rate of 122 mT/m/ms. Conventional sequences, including T1-weighted images (T1WI), T2-weighted images (T2WI), DWI, FLAIR images, and transverse, coronal and sagittal T1WI contrast-enhanced MRI were obtained. MRI parameters are shown in Table 2.

**Table 2:** Description of MRI parameters for Philips Achieva 1.5 T SE MRI systems

	T1WI	T2WI	FLAIR	DWI	Contrast-enhanced^*^
TR (ms)	488	4748	8000	3090	156
TE (ms)	15	110	120	102	2.4
TI (ms)	–	–	2200	–	–
Slice thickness (mm)	6	6	6	6	6
Slice gap (mm)	1	1	1	1	1
Slice number of scanning (layers)	18	18	18	18	20
Scanning time (s)	24	42	24	36	22
NSA	1	2	2	1	2
Field of view (mm^2^)	230 × 201	230 × 201	230 × 183	230 × 201	230 × 199
Matrix	256 × 256	256 × 256	256 × 512	152 × 256	388 × 640

^*^The Contrast-enhanced scan was performed by rapid bolus injection of gadopentate glucosamine (Gd-DTPA) at a dose of 0.1 mmol/kg through the cubital vein at a flow rate of 2.5 ml/s. After bolus injection, 20 mL of saline was intravenously injected. B-values for DWI are 0 s/mm^2^ and 1000 s/mm^2^

### Image and data processing

All measurements were performed on a Philips station. ADC maps were generated from the DWI sequence using Philips station. Image interpretation was done by two radiologists (five years of experience). The investigators were unaware of the patients’ clinical and pathologic information.

The maximum diameter of the lesion was measured on the enhanced images combined with T2WI, T1WI, DWI and ADC images. In the tumor parenchyma region, the region of interest (ROI) was manually selected on the ADC map corresponding to the area of marked enhancement in the contrast-enhanced scan sequence. The ROI was placed in the lesion to select the region significantly enhanced in the contrast-enhanced scan sequence according to T1WI. Adjacent large blood vessels, cystic necrosis, and bleeding areas were avoided. Each lesion was measured three times, and ADC values were the average of 3 measurements. The cystic area after treatment should be included. ADC measurements were performed one week before comprehensive treatment, after one treatment cycle and two treatment cycles. The rates of ADC changes in effective and ineffective groups after one treatment cycle were calculated: ΔADC = (post-treatment ADC-pre-treatment ADC)/pre-treatment ADC × 100%. To measure the maximum diameter of the lesion, we measured the maximum cross-section of the lesion in the contrast-enhanced MRI and expressed it as a mean value of three measurements. If there were controversies between investigators, an average value for ADC was chosen by consensus.

### RECIST

Patients with brain metastases underwent conventional MRI and contrast-enhanced MRI examinations at 1 week before treatment, one treatment cycle and two treatment cycles later. Treatment protocols are listed below. The layer with the maximum tumor diameter was selected; the maximum diameter or the maximum diameter summation of the tumor was measured and used as a grouping standard of the patients. Then the maximum diameters of the tumors 1 week before treatment and two treatment cycles later were compared. According to RECIST criteria, the evaluation of the target lesion is:

Complete remission (CR): the disappearance of almost all target lesions.

Partial remission (PR): the lesions were significantly reduced, and the summation of the maximum baseline diameter was reduced by ≥30%.

Progression (PD): the summation of the maximum baseline diameter increased by ≥20% or new lesions appeared.

Stable (SD): the summation of the maximum baseline diameter reduced but did not reach PR or increased but did not reach PD.

The lesions that met with the CR and PR criteria were identified as effective groups, and the lesions that met the SD and PD criteria were classified as invalid groups.

### Statistical analysis

The data was analyzed by SPSS20.0 software. The measurement data were expressed as mean ± standard deviation (mean ± S.D.). Normal distribution and homogeneity of variance tests were performed on different groups, and the data were in normal distribution. Paired *t*-test was used for comparison between paired samples. The ADC values pre-treatment and one treatment cycle after between the ineffective group and the effective group were compared using the independent sample *t*-test. The receiver operating characteristic (ROC) curve analysis was performed on the degree of change in the average ADC values of the patients treated for one cycle. *P* < 0.05 indicates that the difference was statistically significant.

## Result

### ADC values indicate the tumor progression more sensitively than MRI in brain metastases from lung cancer

**Fig. 1:**
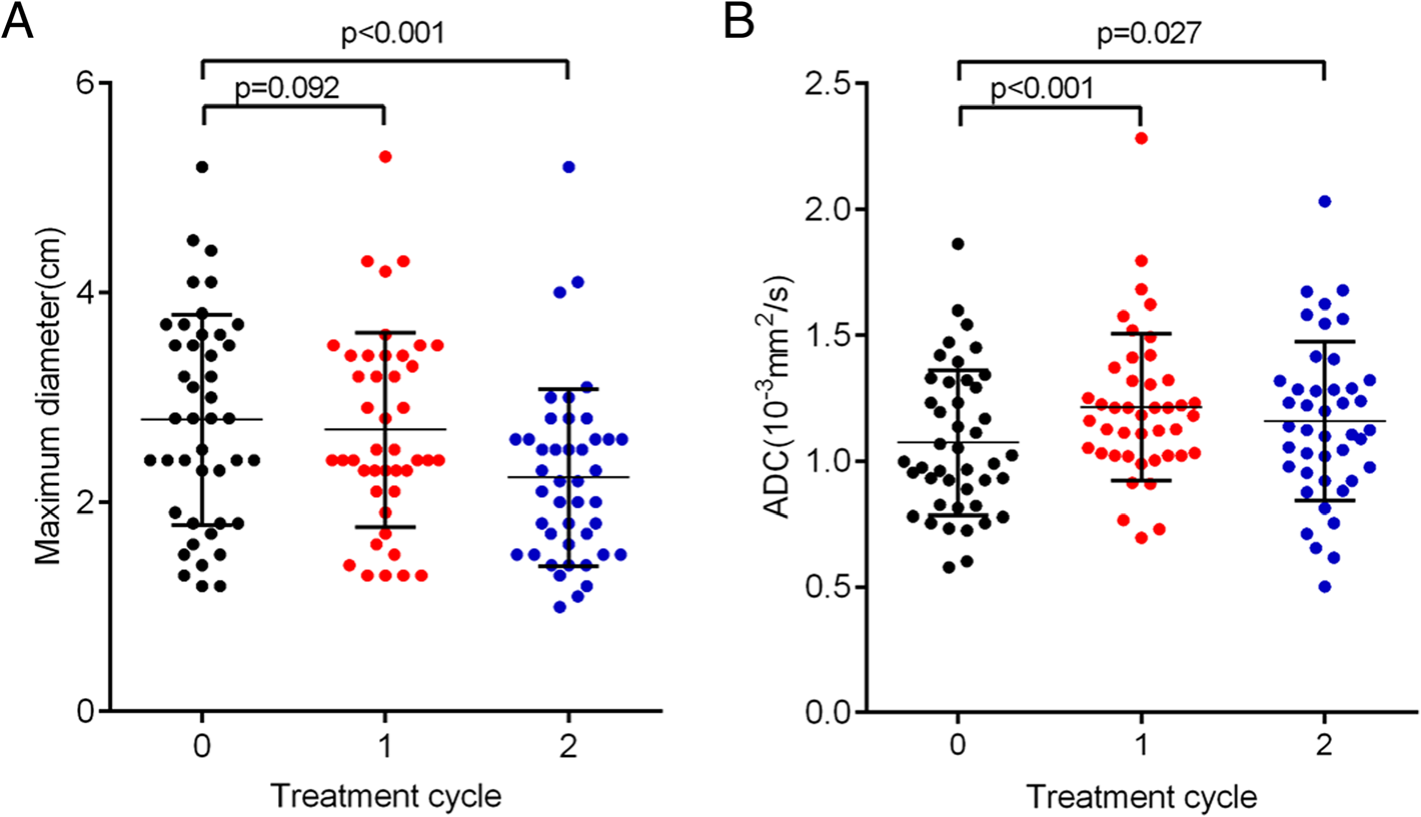
ADC values indicate the tumor progression more sensitively than MRI (**a**) The maximum diameters of tumors for brain metastases from lung cancer before and after treatments. **b** ADC values of brain metastases from lung cancer before and after treatments. *n* = 43, p < 0.05 statistical significance compared to one week before treatment

The maximum diameters of tumors and ADC values of brain metastases from lung cancer patients one week before treatment, one treatment cycle and two treatment cycles later were examined. The results showed that the maximum diameter of the tumor was no difference after one treatment cycle (*p* = 0.092); the maximum diameter of the tumor was significantly decreased after two treatment cycles (*p* < 0.001) (Fig. 1a, Table 3). However, ADC values increased significantly after one and two treatments cycles (Fig. 1b, Table 3), suggesting that ADC values indicate tumor progression more sensitively than the maximum diameters of tumors, especially after one treatment cycle.

**Table 3:** The maximum diameters of tumors and ADC values for brain metastases from lung cancer (*n* = 43)

Treatment	The maximum diameters of tumors (cm)	ADC values (10^−3^ mm^2^/s)
One week before treatment	2.78 ± 0.15	1.07 ± 0.29
One treatment cycle	2.69 ± 0.14	1.21 ± 0.29^**^
Two treatment cycles	2.24 ± 0.13^**^	1.16 ± 0.32^*^

^*^p < 0.05, ^**^p < 0.01, compared to one week before treatment

### ΔADC is a potential monitor of therapeutic efficacy on brain metastases from lung cancer

**Fig. 2:**
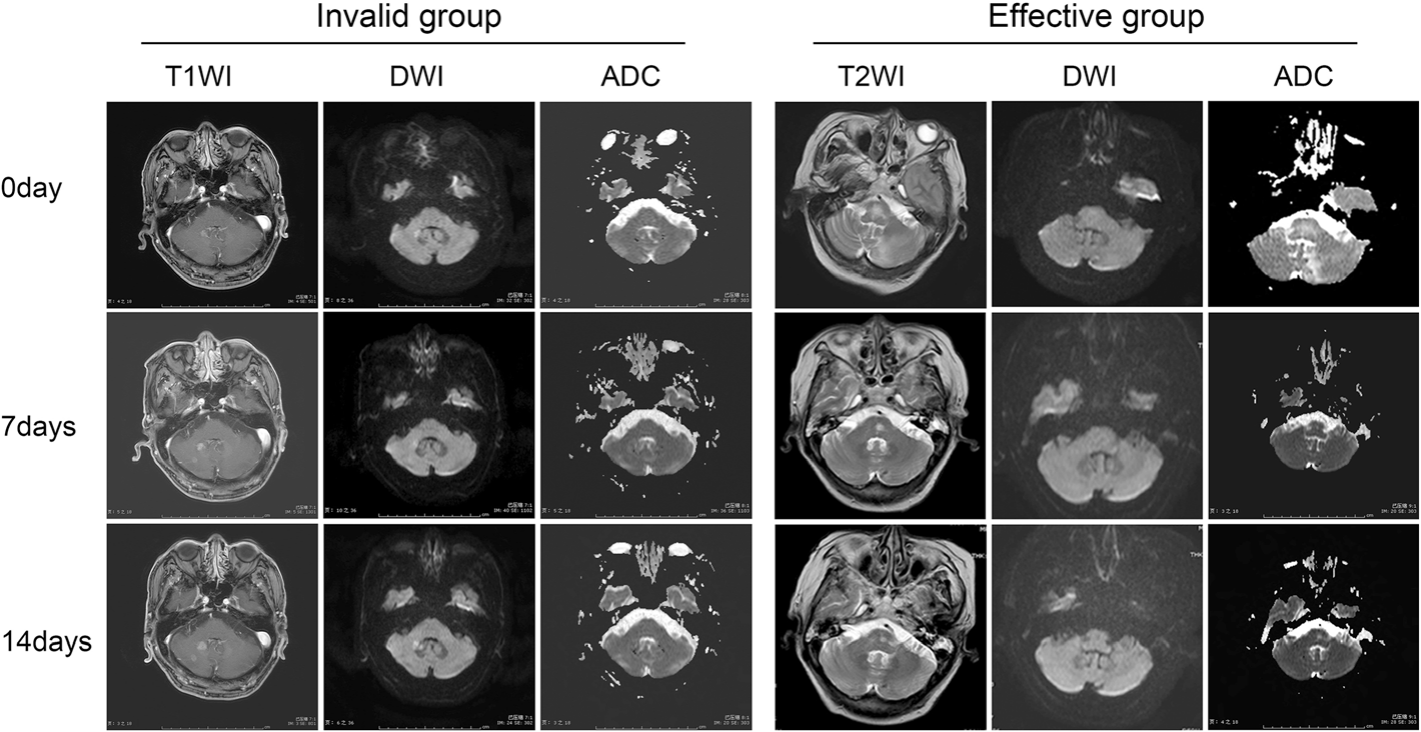
Two cases were shown below for example the group division: Case 1: Pathologically diagnosed as brain metastases from right lung adenocarcinoma. The maximum diameter of the lesion was 10 mm one week before treatment and the ADC value was 0.653 × 10^− 3^ mm^2^/s; the maximum diameter of the lesion was 12 mm and the ADC value was 0.733 × 10^− 3^ mm^2^/s after one treatment cycle; the maximum diameter of the lesion was 14 mm and the ADC value was 0.706 × 10^− 3^ mm^2^/s after two treatment cycles. According to RECIST criteria, the patient belonged to the invalid group. Case 2: pathologically diagnosed as brain metastases from lung cancer. The lesions on the right occipital lobe were nodular with slightly higher signal intensity on T2WI, slightly lower signal intensity on T1WI, high signal intensity on DWI, and obviously enhanced lesions on contrast-enhanced T1WI. The maximum diameter was 17 mm before treatment and the ADC value was 0.759 × 10^− 3^ mm^2^/s; after one treatment cycle, the maximum diameter of the lesion was reduced to about 16 mm, and the ADC value was 1.05 × 10^− 3^ mm^2^/s; after two treatment cycles, the enhanced part of the lesion was obviously reduced, with a diameter of 3 mm, and the ADC value was 1.10 × 10^− 3^ mm^2^/s, suggesting that the treatment was effective. According to RECIST criteria, this patient belongs to the effective group.

Tumor lesions from patients were divided into two groups, effective group (*n* = 22) and invalid group (21), according to RECIST standard (details were shown in Fig. 2), and the ADC was examined. As Table 4 shown, before and after treatment cycles, the ADC values were no statistical difference between the effective group and invalid group. In the effective group, the ADC values were significantly increased after one and two treatment cycles. In the invalid group, the ADC values are not different after one treatment cycle, but decreased after two treatment cycles.

**Table 4:** The change of ADC values after one and two treatment cycles in effective group and invalid group

Treatment	ADC values of effective group (10^− 3^ mm^2^/s)	ADC values of invalid group (10^− 3^ mm^2^/s)
One week before treatment	0.97 ± 0.05	1.17 ± 0.06
One treatment cycle	1.21 ± 0.04^**^	1.20 ± 0.08
Two treatment cycles	1.20 ± 0.05^**^	1.10 ± 0.08^*^

^*^p < 0.05, ^**^p < 0.01, compared to before treatment, no difference of ADC value between effective group and invalid group under different treatment cycles

**Fig. 3:**
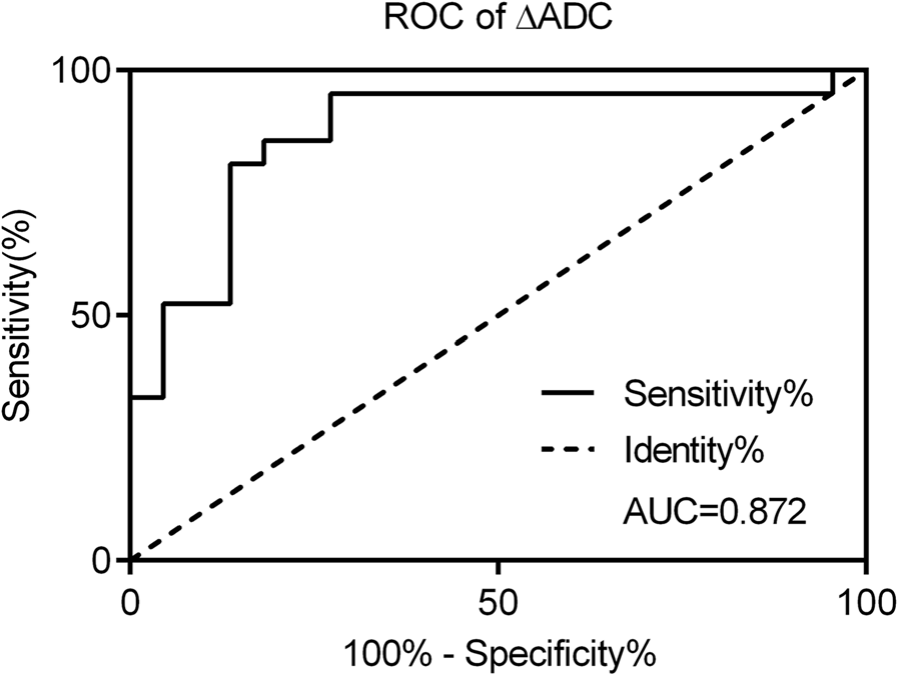
ROC curve analysis of ΔADC values after one treatment cycle. The AUC was 0.872 (sensitivity = 81.8%, specificity = 85.7%)

Then, ΔADC was calculated. As Table 5 shown, after one and two treatment cycles, ΔADC values in effective group were significantly higher than those in the invalid group. ROC curve analysis then revealed that the area under the curve (AUC) of ΔADC after one treatment cycle was 0.872 (sensitivity = 81.8%, specificity = 85.7%, 95% CI: 0.805–0.992) (Fig. 3). These data indicating the potential of ΔADC as a novel index for brain metastasis progression and might help with monitoring therapeutic efficacy and distinguishing effective and invalid groups.

**Table 5:** Comparison of ΔADC values in effective group and invalid group

Treatment	ΔADC values of effective group (%)	ΔADC values of invalid group (%)
One treatment cycle	28.23 ± 3.97^*^	2.5 ± 2.27
Two treatment cycle	27.56 ± 5.16^*^	−7.44 ± 2.62

^*^p < 0.05, compared to invalid group

## Discussion

Brain metastases from lung cancer are common intracranial tumors in adults and the incidence rate is about 20% [16]. With the continuous development of clinical examination methods, the increase in the incidence of lung cancer, and the prolongation of survival after radiotherapy and chemotherapy, the incidence of brain metastases from lung cancer has increased. In the past decades, the whole brain radiation therapy is still the standard treatment for brain metastasis from lung cancer [16], especially for lung cancer with multiple brain metastases.

Nowadays, clinicians commonly evaluate the therapeutic effect ofbrain metastasis from lung cancer by comparing the reduction of tumor size before and after treatment via CT or MRI [17]. Evaluating the therapeutic effect is critical to the further treatment of the patient. In the process of tumor treatment, establishing a method that can evaluate the treatment effect early and objectively becomes a considerable challenge. However, during the course of tumor treatment, the changes of tumor size exist significantly behind the changes in the biological and molecular levels [18, 19]. Due to the different components of the tumor, it is unlikely that different types of tumors will respond to the same treatment [20]. Unreasonable treatment may even accelerate the growth of tumors and causes tumor resistance. Developing the early measurement of biological indicators of therapeutic effectiveness has become an urgent need which can stop ineffective treatment in an early stage and reduce unnecessary toxicity and medical expenses.

Several imaging modalities were wildly applied in cancer diagnosis and assessment of therapy outcome. The tumor vascular physiology and hemodynamics could be measured by perfusion CT (PCT). The PCT parameters were considered as independent predictor of radiation therapy failure in head and neck cancer [21]. Diffusion tensor imaging (DTI) is valuable in diagnosing of idiopathic intracranial hypertension, differentiating gliomas grades and and residual head and neck cancer from post-radiation changes [22–24]. Moreover, combination with arterial spin labeling (ASL) perfusion increased the accuracy of MRI in distinguishing residual/recurrent gliomas from postradiation change [25]. DWI has changed the traditional diagnostic imaging model based on the anatomy and structural changes and led imaging diagnostics to a microscopic molecular level. It is currently the only non-invasive detection of water molecules free diffusion movement in vivo. The ADC value of the water molecules is determined by the viscosity of the molecules, the permeability of the cell membrane, the direction of the tissue, and the structure of the cells that impede the movement of the water molecules [26, 27]. Therefore, the ADC values can distinguish tumor cells from non-cellular regions, cystic regions from solid regions, and the critical issues at the cellular level during tumor therapy. Recently, both animal and cell models have demonstrated that ADC values are of great importance in prognosis and the early monitoring of tumor therapeutic efficiency [28–30]. For example, lower ADC values were associated with greater tumor size and highly aggressive in cerebral cancers [31]. In addition, there are few human studies in this area, including gliomas [32], breast tumors [33], hepatic tumors [34], and rectal tumors [35], and all obtained the similar results.

Previous studies revealed that a higher ADC mean showed a longer overall survival regardless of adjuvant therapies in non-small cell lung cancer cerebral metastasis [36]. In the present study, we revealed that the significant increase of ADC values was appeared after one treatment cycle, which was earlier than the presence of tumor maximum diameter alterations (after two treatment cycles). Effective anti-tumor therapy results in necrosis of tumor cells, reduced cell density, the disappearance of cell membrane integrity, increased extracellular space, and thus increased ADC values which can be sensitively detected by DWI [37]. Therefore, by observing the movement of water molecules in the tumor, the ADC value can be used as an early indicator to evaluate the effect of tumor treatment in brain metastasis from lung cancer.

Tumors with higher ADC values often contain more necrotic tissue and/or damaged cell membranes, with a poorer blood perfusion, and are relatively insensitive to radiotherapy [38], indicating that pre-radiotherapy ADC values can predict radiotherapy effects to a certain extent and can be used by clinicians to assess the sensitivity of tumors before radiotherapy. However, in the present study, no statistical difference was noted between effective group ADC values and invalid group ADC values before treatment cycle. In addition, we demonstrated that the ΔADC values were significantly higher in effective group than those in the invalid group. The AUC of ΔADC is 0.898 with 81.8% sensitivity and 85.7% specificity. These results demonstrated that ΔADC values have a potential to monitor therapeutic efficacy and distinguish chemoradiotherpy sensitive and insensitive brain metastasis from lung cancer.

There are a few limitations of this study. First, the small number of patients might limit the statistical results of the cases. Second, there is lack of long time follow-up of patients after treatment. The correlation of ADC with the overall survival in patients with brain metastasis from lung cancer is needed in the further studies.

## Conclusions

In summary, ADC value examination during the chemoraidotherapy treatment cycles can help early monitoring and dynamic observation of therapeutic efficacy in brain metastases from lung cancer. It is possible for clinicians to quickly, accurately and non-invasively predict and monitor the responses of tumors before and during treatment, to stop ineffective treatment early and to reduce unnecessary toxicity and medical expenses.

## Abbreviations

ADC: Apparent diffusion coefficient
ASL: Arterial spin labeling
AUC: The area under the curve
CABG: Coronary Artery Bypass Grafting
CR: Complete remission
DTI: Diffusion tensor imaging
DWI: Diffusion weighted imaging
PCT: Perfusion CT
PR: Partial remission
ROC: The receiver operating characteristic
T1WI: T1-weighted images
T2WI: T2-weighted images
